# Synapto-Protective Drugs Evaluation in Reconstructed Neuronal Network

**DOI:** 10.1371/journal.pone.0071103

**Published:** 2013-08-16

**Authors:** Bérangère Deleglise, Benjamin Lassus, Vaneyssa Soubeyre, Aurélie Alleaume-Butaux, Johannes J. Hjorth, Maéva Vignes, Benoit Schneider, Bernard Brugg, Jean-Louis Viovy, Jean-Michel Peyrin

**Affiliations:** 1 Neurobiologie des Processus Adaptatifs, CNRS, UMR7102, Paris, France; 2 Université Pierre et Marie Curie, Paris, France; 3 Inserm UMR-S 747, Paris, France; 4 Université Paris Descartes, Sorbonne Paris Cité, UMR-S 747, Paris, France; 5 Macromolécules et Microsystèmes, CNRS UMR168, Institut Curie, Paris, France; 6 Integrative Neurophysiology, Center for Neurogenomics and Cognitive Research (CNCR), VU University Amsterdam, Amsterdam, The Netherlands; Macquarie University, Australia

## Abstract

Chronic neurodegenerative syndromes such as Alzheimer’s and Parkinson’s diseases, or acute syndromes such as ischemic stroke or traumatic brain injuries are characterized by early synaptic collapse which precedes axonal and neuronal cell body degeneration and promotes early cognitive impairment in patients. Until now, neuroprotective strategies have failed to impede the progression of neurodegenerative syndromes. Drugs preventing the loss of cell body do not prevent the cognitive decline, probably because they lack synapto-protective effects. The absence of physiologically realistic neuronal network models which can be easily handled has hindered the development of synapto-protective drugs suitable for therapies. Here we describe a new microfluidic platform which makes it possible to study the consequences of axonal trauma of reconstructed oriented mouse neuronal networks. Each neuronal population and sub-compartment can be chemically addressed individually. The somatic, mid axon, presynaptic and postsynaptic effects of local pathological stresses or putative protective molecules can thus be evaluated with the help of this versatile “brain on chip” platform. We show that presynaptic loss is the earliest event observed following axotomy of cortical fibers, before any sign of axonal fragmentation or post-synaptic spine alteration. This platform can be used to screen and evaluate the synapto-protective potential of several drugs. For instance, NAD^+^ and the Rho-kinase inhibitor Y27632 can efficiently prevent synaptic disconnection, whereas the broad-spectrum caspase inhibitor zVAD-fmk and the stilbenoid resveratrol do not prevent presynaptic degeneration. Hence, this platform is a promising tool for fundamental research in the field of developmental and neurodegenerative neurosciences, and also offers the opportunity to set up pharmacological screening of axon-protective and synapto-protective drugs.

## Introduction

In chronic neurodegenerative syndromes such as Alzheimer’s and Parkinson’s diseases, or acute syndromes such as ischemic stroke or traumatic brain injuries, neuronal degeneration proceeds through a protracted dying-back pattern in which dysfunction of nerve terminals precedes neuronal cell body destruction [1], [2], [3]. Progressive loss of synaptic connections leads to the lack of controlled synaptic transmission, generation of abnormal neurotransmission, and a reduced level of trophic factors, resulting in impairment of postsynaptic neuronal survival and therefore progressive weakening of the robustness of interconnected networks. These events may account for early cognitive symptoms in patients [4], [5]. Synapse failure thus plays a critical role in the initiation and progression of many neurodegenerative conditions [6], [7], [8], [9]. Moreover, as evidenced by Braak and co-workers, degeneration associated with Alzheimer’s or Parkinson’s diseases follows specific neuronal pathways in the brain [6], [7], [8], [9], [10], [11], [12]. Together, these observations highlight the need to study neurodegenerative processes at the neuronal network level [13], [14], [15]. Synaptic degeneration being an early and seminal process in various acute and chronic degenerative syndromes, it is important to develop efficient neuroprotective strategies that target molecular mechanisms involved in synaptic collapse.

The human brain is a complex organ composed of (∼200) billion cells and even more complex interconnected cellular interactions. Current experimental models used to study synaptic stability under pathological conditions range from whole animal models that preserve the anatomical structures but greatly limit experimentation at cell level, to dissociated cell culture systems that allow detailed manipulation of cell phenotypes but lack the highly ordered and instructive brain environment. In previous work we and others demonstrated that microfluidic technologies for cell culture are powerful tools allowing exquisite control of the neuronal micro-environment and offering the possibility to reconstruct fully functional neuronal pathways *in vitro* [16], [17], [18], [19], for review see [20], [21], thus paving the way to study neurodegenerative syndromes on chips. In the present study we present a microfluidic platform allowing axotomy of reconstructed neuronal pathways and show that chemical axotomy of cortical fibers triggers a rapid presynaptic disconnection from striatal dendrites. Our synaptic damage assay recapitulates the molecular events previously mapped during *in vivo* experiments. We perfused pharmacological compounds targeting specific molecular pathways in the synaptic compartment, and used automated image analysis quantification of presynaptic collapse. We demonstrate that a rho Kinase inhibitor (Y27632), and nicotinamide adenine dinucleotide (NAD^+^), exert strong synapto-protective activities whereas zVAD-fmk and resveratrol fail to protect synapses. We propose the use of this sophisticated “brain on chip” as a versatile platform for fast evaluation of synapto-protective drugs.

## Material and Methods

### Chip Design and Master Production

**Figure 1:**
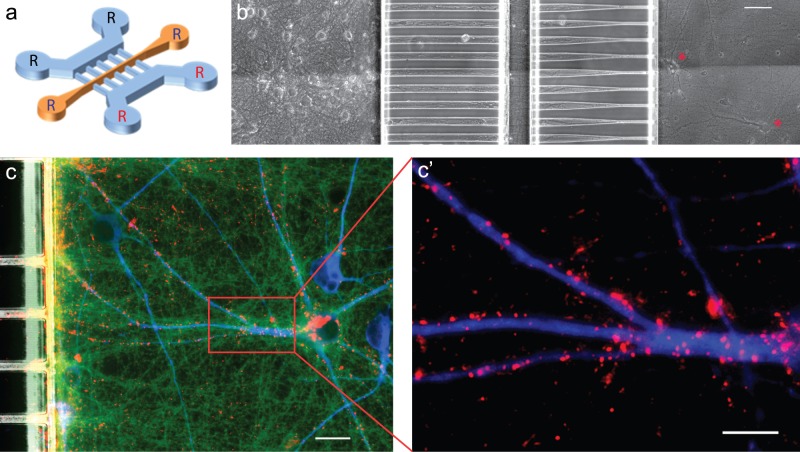
Reconstruction of oriented neuronal networks in 3C-microfluidic chip. **a**: Microfluidic neuronal culture devices are made up of two separate cell culture chambers (blue) interconnected by a series of asymmetrical micro-channels interrupted by a central narrow (50 µm-wide) channel that gives access to the central part of the axons. Each chamber is perfused individually by two reservoirs (indicated by R). **b**: Phase contrast image of a reconstructed oriented neuronal network in 3C-chip. Cortical neurons are seeded in the left chamber and connect striatal neurons seeded in the right chamber (red stars). The size of asymmetrical micro-channels allows the outgrowth of axons but prevents the entry of cell bodies; micro-channels allow the passage of axons only in a left-to-right direction (axonal diodes) *scale bar = 50* *µm*. **c–c’**: Immunofluorescent images of the receiving (right) chamber. Cortical axons exit micro-channels (green: α-tubulin) and cluster presynaptic markers (red: v-GLUT1) on striatal dendrites (blue: MAP-2). **c’**: Enlargement of the clustering of cortical presynaptic labeling on striatal dendrites, suggesting cortico-striatal synapse establishment. *scale bar = 20 μm.*

Microfluidic channels are made up of two elements: 55 µm high macro-chambers for cell or fluid injection, separated by narrowing arrays of 3 µm high micro-channels allowing directional axonal outgrowth. The three compartmented chip (“3C”) was constructed using the following design: two rectangular macro-channels (length 4000 µm, width 500 µm, height 55 µm) separated by arrays of asymmetrical micro-channels (length 500 µm, width 15 to 3 µm, height 3 µm, [17]) interrupted by a third macro-channel inserted in the middle of the micro-channel “diode” array (see Fig. 1a,b). This third macro-channel acts as an intermediate compartment allowing control of the flow of solution over the mid-portion of cortical axons [16].

To produce the template with elements of two different thicknesses, we used two layers of photoresist (SU82005 and SY355). A silica wafer was dehydrated by heating it to 150°C for 10 minutes and activated by plasma treatment (30s). A first layer of SU82005 (Microchem) was spin-coated onto the wafer at 3800 rpm and then soft-baked at 65°C for 2 minutes and 95°C for 4 minutes. The template was then exposed to UV light through an optic mask (made of plastic or quartz if the required resolution was less than 8 µm). After hard bake, the channels (3–4 µm high) were developed in SU8 developer. The injection channels were then produced by laminating the newly obtained wafer with a 55 µm-thick layer of dry film SY355 (Elga Europe) at 90°C. A second mask was aligned over the SU8 channels and after exposure to UV light, the channels were baked for 10 minutes at 120°C and developed in BMR developer, then rinsed with BMR rinse, acetone and isopropanol.

The quality of the narrowing micro-channels was assessed by white-light optical profiling (WLOP) in vertical scanning mode (VSI), with an interference microscope Wyko® NT1100 (Veeco Instruments Inc., Plainview, NY, USA); this is a non-contact optical profiling system, based on light interferences, that provides high vertical resolution.

### Microfluidic Chip Production

Polydimethylsiloxane (Sylgard 184, PDMS, Dow Corning, Midland, MI, USA) was mixed with a curing agent (9∶1 ratio) and degassed under vacuum. The resulting preparation was poured onto a polyester resin replicate and reticulated at 70°C for 2 hours. The elastomeric polymer print was detached and two reservoirs were punched for each macro-channel. The resulting piece was cleaned with isopropanol and dried. The polymer print and a glass cover slip were treated for 200 seconds in an air plasma generator (98% power, 0.6mBar, Diener Electronic, Ebhausen, Germany) and bonded together. The chips were placed under UV for 15 minutes and then coated with a solution of poly-D-lysine (10 µg/mL, Sigma; St. Louis, MO, USA) overnight and washed with PBS before cell seeding.

### Primary Neuronal Cultures

Animal care was conducted in accordance with the standard ethical guidelines of the CNRS “Formation à l’Expérimentation Animale” and approved by the “C2EA - 05 Comité d'éthique en expérimentation animale Charles Darwin”. Timed pregnant mice at E14 were purchased from René Janvier and cared for by the well-established animal care facility at University Pierre et Marie Curie (IFR83). Pregnant mice were decapitated and cortices and striata were micro-dissected from E14 embryos of Swiss mice (Janvier, Le Genest Saint Isle, France) and of a transgenic mice strain expressing GFP (green fluorescent protein) under the control of an actin promoter in all cell types [22]. All steps of dissection were in cold phosphate buffer saline (PBS) supplemented with 0.1% glucose (Life Technologies, Inc., Gaithersburg, MD, USA). Dissected structures were digested with trypsin-EDTA for striata (Life Technologies, Inc., Gaithersburg, MD, USA) or papaïn for cortices (20U/ML in DMEM, Sigma; St. Louis, MO, USA). After tryspin or papaïn inactivation with fetal bovine serum (FBS, PAA Piscataway, NJ, USA), structures were mechanically dissociated with a pipette in presence of DNAse. After several rounds of rinsing, cells were re-suspended in DMEM (Life Technologies, Inc., Gaithersburg, MD, USA), to a final density of 45 million cells/ml for cortices and 12 million cells/mL for striata. Cortical cells were then seeded in the somatic compartment and striatal cells in the distal compartment: 3 µl of the cell suspension was introduced into the upper reservoir and cells flowed into the chamber and adhered within 1–2 minutes. Cell culture medium was then added equally to the four reservoirs (60 µl/reservoir). Both neuronal cell types were grown in DMEM glutamax+streptomycin/penicillin (Life Technologies, Inc., Gaithersburg, MD, USA) +10% FBS+N2+ B27 (Life Technologies, Inc., Gaithersburg, MD, USA). Microfluidic chips were placed in plastic Petri dishes containing H_2_O-EDTA to prevent evaporation and incubated at 37°C in a humid 5% C02 atmosphere. The culture medium was renewed every six days. Upon differentiation, 2 or 3 days after seeding, cortical axons entered the micro-channels and reached the second chambers after 5 to 6 days. Cortical axons continued growing thereafter.

### Pharmacological Treatment

All chemicals were prepared as concentrated solution according to the recommendations of the different manufacturers. Compounds were aliquoted in Eppendorf tubes and used once to avoid repeated freezing/thawing processes. Aliquots were stored at −80°C for no longer than two months. Care was taken to protect photosensitive molecules from light by wrapping the test tubes in aluminum foil. Drugs were extemporaneously diluted at their respective final concentration in DMEM containing 10% SVF+N2+ B27.

For pharmacological pretreatments, medium was removed from the striatal chambers and was replaced by DMEM containing the pharmacological drugs. Except for NAD^+^, which was administrated overnight, pharmacological compounds were perfused one hour before axotomy. The following concentrations were used for control or axotomy conditions: resveratrol (20 µ*M,* Sigma; St. Louis, MO, USA), z-VAD (50 µ*M,* Sigma; St. Louis, MO, USA), Y27632 (10 µ*M,* Sigma; St. Louis, MO, USA) or NAD^+^ (5 m*M,* Sigma; St. Louis, MO, USA). After axotomy of the central channel, fresh medium containing drugs was applied to wash detergent.

### Live Imaging

Live imaging of striatal dendritic spines was carried out using a transgenic mouse expressing GFP under the control of an actin promoter [22]. Non-transgenic cortical neurons were seeded in the emitting chamber, while striatal neurons from the GFP-expressing mouse were cultivated in the receiving chamber. Fluorescent live imaging of striatal dendritic spines was carried out in control or axotomized conditions for 6 hours using a 63X oil-objective.

### Immunofluorescence

At various times, cultures were fixed in 4% paraformaldehyde (PFA, Sigma; St. Louis, MO, USA) for 20 minutes at room temperature. Cells were then washed twice with PBS for 5 minutes and permeabilized for 45 minutes with 0.2% Triton X-100 and 1% BSA in PBS. Primary antibodies were then added and the samples incubated at 4°C overnight in PBS. The samples were rinsed twice for five minutes with PBS and further incubated with the corresponding secondary antibodies for two hours at room temperature. The chips were then rinsed once with PBS and once with PBS+0.1% sodium-azide, and mounted in moviol mounting medium. The following primary antibodies from Sigma (Sigma; St. Louis, MO, USA) were used: α-tubulin-FITC (monoclonal 1/700); Microtubule Associated Protein -2 (MAP-2, mouse monoclonal 1/500); Synaptophysin (mouse monoclonal 1/500); vesicular glutamate transporter 1 (v-GLUT1, rabbit polyclonal 1/500, gift E. Herzog, CNRS, UMR 7224). Species-specific secondary antibodies coupled to Alexa 350, 488, or 555 were used (1/500, Life Technologies, Inc., Gaithersburg, MD, USA) to visualize bound primary antibodies.

### Neuronal Network Axotomy

After full maturation of the neuronal network (14 days after seeding), cell culture medium was removed from the two reservoirs connecting the central chamber, and solution of plain DMEM (sham) or 0.1% Triton in DMEM was flowed from the upper reservoir for 30 seconds. Careful attention was paid to the fluidic isolation of the central channel: the two outer cell culture chambers were pressurized with 50 µl of cell culture medium while 20 µl of sham or detergent-containing medium were introduced into the upper reservoir of the central channel. This created a flux of detergent into the central chamber containing the cortical axons. The central chamber was then washed three times with DMEM and the central reservoirs were then filled with medium. Axotomized cells were then kept at 37°C under 5% CO_2_ for various periods before fixation.

### Image Acquisition

Images were acquired with an Axio-observer Z1 (Zeiss, Germany) fitted with a cooled CCD camera (CoolsnapHQ2, Ropert Scientific). The microscope was controlled with Metamorph software and images were analyzed using ImageJ and SynD software [23].

### Quantification of Synaptic Disconnection

Synaptic disconnection was assessed by counting v-GLUT1 clusters affixed to MAP-2 positive striatal dendrites. All images were obtained using the same acquisition parameters. The images were similarly processed with ImageJ software before being used for quantification: the brightness/contrast of all control images was optimized manually to eliminate the background and to maximize the signal. The means of the minimum and maximum intensities were then calculated in the control condition and these settings were applied to all images. The images of the three stainings v-GLUT1/α-Tubulin/MAP-2 were merged and the resulting image was used to define the zone where striatal dendrites were sufficiently innervated by cortical fibers. A v-GLUT1/MAP-2 merge was then used for quantification using SynD software (Fig. S1). The merged v-GLUT1/MAP-2 images (Fig. S1A) were analyzed using SynD [23]. Here the somata were detected by thresholding the MAP-2 channel, and smaller structures such as neurites were removed by morphological erosion followed by dilation (Fig. S1B). Neurites were detected using steerable filters applied to the MAP-2 channel (Fig. S1C). Finally, synapses were detected by thresholding the v-GLUT1 channel to get a synapse mask (Fig. S1D) and then putative individual synapse centers were detected by deconvolving the original v-GLUT1 channel and locating peaks (Fig. S1E). Only synapses detected on or near the neurites were included for analysis. The resulting synapse counts were then exported to Excel for further analysis. The settings used were: Soma erode = 25; Morphology threshold = 35; Max cost = 0.9; Steerable filter size (in *µm*) = 1.6; Synapse intensity threshold in standard deviations above noise = 0.3; Neurite padding (zone in *µm* outside neurite mask where synapses are counted) = 8; Minimal size = 0.3 *µm.* Reported values are means for at least three independent experiments, each performed in triplicate.

### Statistical Analysis

For synapse quantification, differences were assessed by ANOVA, followed, when appropriate, by a post-hoc Bonferoni test. For all analyses: *p-value<0.05; **p-value<0.01; ***p–value<0.001.

## Results

### Setting up a Microfluidic Cell System to Model Axonal Injury in Oriented Neuronal Networks

To assess the impact of axonal injury on distant presynaptic dynamics we designed a microfluidic platform allowing the manipulation of reconstructed neuronal networks encompassing three separate sub-compartments (Fig. 1). This was achieved by combining two approaches previously developed in our laboratories. The first system was designed to allow easy axotomy of neuronal axons in a three-compartment chip presenting a somato-dendritic compartment, a narrow central channel giving access to the central part of the axons, and a distal receiving chamber containing axons [16].The second system comprised asymmetrical micro-channels to polarize axonal growth and reconstruct oriented neuronal networks *in vitro* in a two-compartment chip [17].

The new device is composed of a three-compartment chip in which the diode array is interrupted by a 50 µm-wide central channel (Fig. 1a and b).The system is made up of two distinct cell culture chambers (for each neuron type), each connected to two reservoirs (indicated by an R in Fig. 1a) and separated by an array of 500 µm-long narrowing micro-channels allowing unidirectional axonal growth (from the proximal chamber to the distal one) and interrupted by a third central channel giving access to the central part of the axons (orange channel in Fig. 1a). Using this multi-compartment device, we next reconstructed an oriented cortico-striatal neuronal network in which the somatic, axonal and synaptic compartments of cortical neurons are specifically accessible through fluidic perfusion. To that purpose, mouse primary cortical neurons were seeded in the left chamber (on the wider side of the narrowing micro-channels), and striatal neurons from the same mouse were seeded in the receiving chamber. Fig. 1b shows phase contrast micrographs of cortical neurons projecting towards striatal neurons. Neurons seeded in the chamber with large micro-channel openings emit axons that invade the micro-channels and grow within these tubes. When exiting the first array of micro-channels, about 80% of the cortical fibers crossed the central chamber, reached the second array of narrowing micro-channels and continued to grow to connect striatal neurons seeded in the receiving cell culture chamber (Fig. 1b). As described previously [17], due to the small size of the micro-channel tips, axons from the receiving striatal chamber bumped into the sidewall of the chamber and did not shoot towards the cortical chamber. Fifteen days after cell seeding, the neuronal network was fully established and cortical fibers were connected to fully differentiated striatal neurons, as evidenced by the docking of VGLUT1 positive presynaptic cortical terminals to MAP-2 positive striatal dendritic arborization (Fig. 1c’) and the presence of GFP positive dendritic spines on striatal dendritic shafts. Connected striatal neurons showed slow spontaneous calcium oscillation imposed by cortical spontaneous rhythms (data not shown and [17]). Overall, introducing a central chamber dedicated to the manipulation of the mid portion of cortical fibers did not modify neuronal differentiation in reconstructed networks.

### Kinetics of Structural Alterations after Cortical Fiber Axotomy

**Figure 2:**
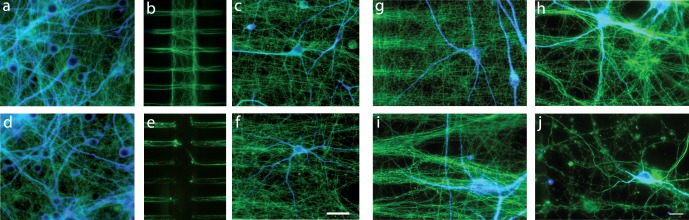
Kinetics of axonal degeneration following cortical axotomy in a network. **a,d**: Cortical, **b,e**: central and **c,f**: striatal, chambers of normal (**a–c**) or axotomized (**d–f**) reconstructed cortico-striatal networks (div14). Neurons were stained for MAP-2 (blue), α-tubulin (green) and v-GLUT1 (red). **a–c**: Cortical neurons send axons efficiently through the micro-channels and the central chamber to reach, and connect with, striatal neurons. **d–f**: Two hours after axotomy, no axons are present in the central chamber but axons inside the micro-channels and in the striatal chamber remain intact. *scale bar = 75* *µm* **g–j**: Kinetics of degeneration of the distal part of the cortical axons following axotomy of cortical fibers in the receiving chamber. No axonal fragmentation (assessed by α-tubulin, green) is observed either in non-axotomized condition (**g**), or two hours after axotomy (**h**). The first tubulin beads (blebs) on cortical axons appear four hours after axotomy (**i**), and after six hours, all cortical axons disconnected from their soma are fragmented whereas striatal morphology remains intact (MAP-2, bleu) (**j**). *scale bar = 20* *µm.*

Synaptic and axonal degeneration are two of the earliest structural alterations occurring in various acute and chronic neuronal pathologies, and often precede the destruction of the cell body. Severing of peripheral or central nerves is widely used as an experimental model of brain and axonal trauma in order to decipher specific molecular pathways involved in axonal degeneration. However, the molecular mechanisms and kinetics involved in presynaptic breakdown of nerve terminals remain elusive. In order to assess the impact of axonal trauma on synaptic terminals we axotomized the reconstructed cortico-striatal neuronal network. Axotomy was carried out by perfusing cell culture medium containing 0.1% Triton for 30 seconds in the central compartment containing cortical axons (Fig. 2a–f). As shown in Fig. 2e we monitored minute severing of cortical axons within the central channels without damaging the proximal parts of the fibers within the micro-channels. We next performed a kinetic analysis of neuronal network component degeneration. The cortical somato-dendritic compartment showed no signs of either tubulin architectural alterations or dendritic alteration following axotomy (MAP-2 and α-tubulin staining, Fig. 2a,d). The portion of axon still connected to the cortical soma was able to re-grow after axotomy (data not shown), thus indicating that axotomy of cortical fibers does not trigger a retrograde death signaling process. Compared to sham perfused fibers (Fig. 2g), severed cortical axons in the striatal chamber (Fig. 2h) displayed no signs of axonal tubulin fragmentation even two hours after axotomy. Axonal degeneration started four hours after lesion (i) with sparse tubulin beading (axonal blebs); six hours after axotomy all axons were fragmented (j). Meanwhile, the global architecture of the striatal dendritic tree (revealed by MAP-2 labeling) remained globally unaffected during the first six hours.

**Figure 3:**
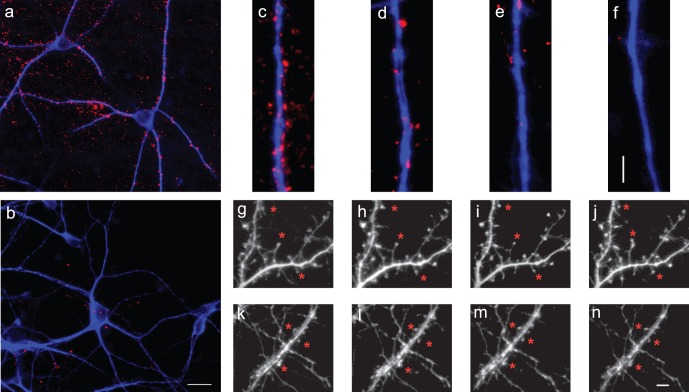
Early cortico-striatal synaptic disconnection after cortical fiber axotomy. **a–f**: Cortical presynaptic structures (v-GLUT1, red) affixed to striatal dendrites (MAP-2, blue) in control conditions (**a**), three hours after axotomy (**b**)*scale bar: 20* *µm.* Note the disappearance of v-GLUT1 labeling suggesting cortical presynaptic degeneration. **c–f**: Representative high magnification images in control conditions (**c**) and two hours (**d**), four hours (**e**) and six hours (**f**) after axotomy. Note the fast cortico-striatal disconnection as soon as two hours after axotomy. *scale bar: 5* *µm.* **g–n**: Live imaging of GFP-expressing striatal neurons at time 0h (**g,k**), 2h (**h,l**), 4h (**i,m**) and 6h (**j,n**) in control (**g–j**) or axotomy (**k–n**) conditions. Note the stability of striatal dendritic spines (arrow) in both conditions. s*cale bar: 15* *μm.*

The number of cortical v-GLUT1 clusters on striatal dendrites was counted by widefield epifluorescence microscopy at various times after axotomy. Under control conditions (Fig. 3a,c), there were approximately 40 v-GLUT1presynaptic terminals per 100 µm of striatal dendrite. Two hours after axotomy, the number of presynaptic cortical clusters on striatal fibers was 60% lower (Fig. 3d) than in control cultures (Fig. 3c), whereas cortical axons showed no signs of blebbing or disorganization of their microtubular network. Four hours after axotomy (when the first sign of axonal alteration appeared), presynaptic density was 75% lower in axotomized conditions (Fig. 3e) compared to sham experiments (Fig. 3a,c). Finally, six hours after axotomy, when the cortical axons were almost all fragmented (Fig. 2j), presynaptic v-GLUT1 staining had completely disappeared (Fig. 3f), indicating complete destruction of cortico-striatal synapses. We concluded that cortical axotomy triggers a progressive cortico-striatal synaptic disconnection, which precedes the fragmentation of cortical axons.

As shown in Fig. 2g–j, the global architecture of striatal neurons did not seem to be altered during the axotomy time course. However, we wondered whether presynaptic cortical degeneration would induce a milder postsynaptic alteration. Non-transgenic cortical neurons were seeded in the emitting chamber, while striatal neurons from a transgenic mouse expressing GFP (green fluorescent protein) under the control of an actin promoter were grown in the receiving chamber. Fluorescent live imaging of striatal dendritic spines was carried out in control or axotomized conditions for six hours (Fig. 3g–n). In control conditions, striatal spines were stable (Fig. 3g–j). Following cortical fiber axotomy the striatal spines remained stable even when cortical pre-synapses previously connecting those spines had almost all degenerated (Fig. 3k–n). These observations suggest that presynaptic disorganization represents one of the primary events induced by axonal damage, which in turn induces the anterograde propagation of a fast degenerative signal within the axons.

The loss of presynaptic clusters was also associated with the apparent dispersal of the residual v-GLUT1 vesicles (not shown). There are several possible explanations for this observation. First, v-GLUT1-positive vesicles may disperse along axon fibers due to local modification of the mechanical tension of axonal fibers triggered by axotomy. Second, dispersion of v-GLUT1 and presynaptic proteins may be associated with the collapse of the whole structure of the spine. It would be of interest to monitor the dynamics of v-GLUT1 movement, using v-GLUT1-GFP chimeric protein, to address this issue.

Live imaging of striatal dendritic spines revealed that striatal spines did not degenerate within the same time-window as pre-synapses (compare Fig. 3g–n with Fig. 3a–f). Even four hours after axotomy, when almost all the synapses had degenerated and axons started to fragment, striatal postsynaptic architecture remained intact (Fig. 3m). This suggests that structural postsynaptic integrity can be preserved for at least four hours after the loss of presynaptic structures.

### Pharmacological Evaluation of Synapto-protective Drugs in 3C-microfluidic Chip

Using the above data it was possible to identify a time-window within which pre-synapses had almost all degenerated after cortical axotomy while axons or postsynaptic elements remained intact. This time-window can be used to screen for drugs expected to have the capacity to slow down or prevent synaptic loss.

**Figure 4:**
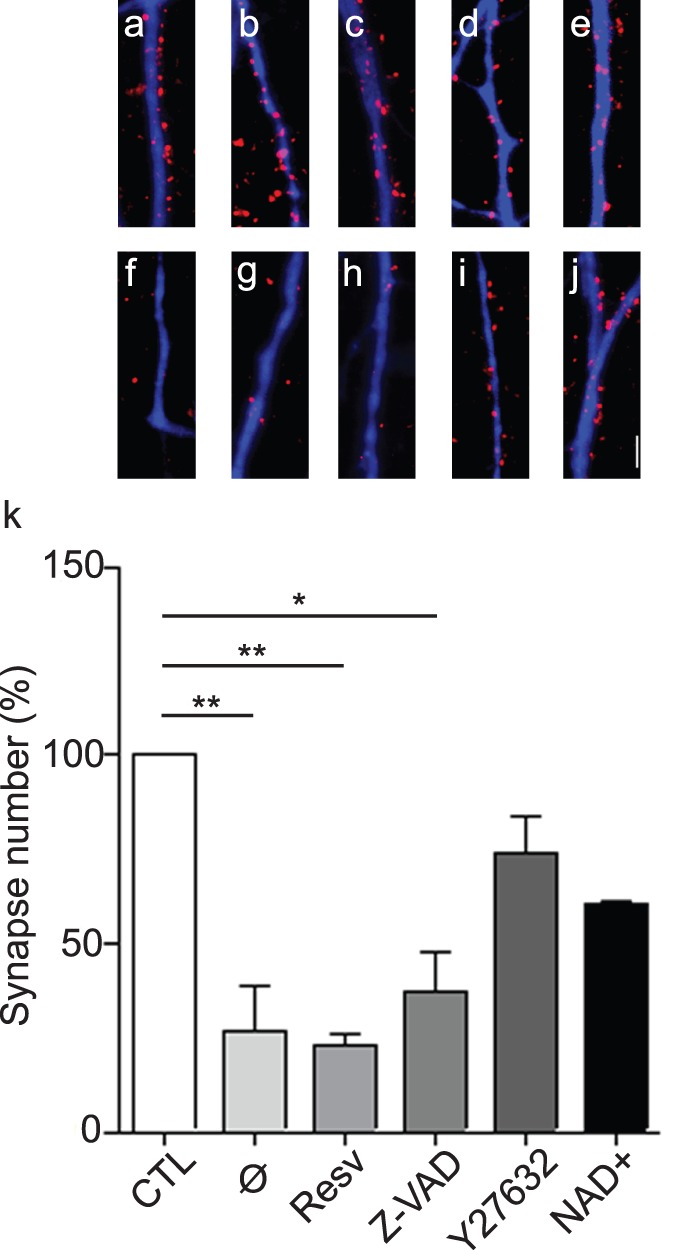
Pharmacological screen of synapto-protective drugs in 3C-microfluidic chip. **a–j**: Representative high magnification images of cortical presynaptic structures (v-GLUT1, red) affixed to striatal dendrites (MAP-2, blue) in non-axotomized (**a–e**) and 3 hours post-axotomy (**f–j**) conditions, without treatment (**a,f**), or with resveratrol (20 µ*M* **b,g**), 50 µ*M* z-VAD (**c,h**), 10 µ*M* Y27632 (**d,i**) or 5 m*M* NAD^+^ (**e,j**) pre-treatment. *scale bar: 5* *µm*. **k**: Quantification of cortical presynaptic structures affixed to MAP-2-positive striatal dendrites after cortical axotomy. The graph represents the relative number of synapses remaining compared to non-axotomized condition. Y27632 and NAD+ delay cortical synaptic loss whereas no significant protection is observed with resveratrol or z-VAD pre-treatment.(ANOVA 2, *p-value<0.05, **p-value<0.01). Synaptic degeneration was quantified using SynD software (see Figure S1) which allows automatic segmentation of striatal dendritic trees and co-localization of VGLUT1 positive synapses docked to the dendrites [23].

With the help of our microfluidic-based synaptic stability/degeneration assay, we first quantified synaptic degeneration using SynD software (see [23] and Fig. S1), which allows automatic segmentation of striatal dendritic trees and co-localization of v-GLUT1 positive synapses docked to the dendrites. Automatic counting was used to evaluate the number of v-GLUT1 clusters on striatal dendrites in control conditions (Fig. 4a–e) or three hours after axotomy (Fig. 4f–j), when cortical pre-synapses have normally degenerated (but not cortical axons or striatal spines).

We then selected several pharmacological compounds known to impede synaptic and/or axonal degeneration *in vivo.* Although the precise molecular events triggered in synaptic destruction upon neurodegenerative stimuli are still unknown, previous experiments conducted after *in vivo* axotomy of peripheral nerves have shown that presynaptic collapse at the neuromuscular junction is triggered by a caspase-independent, NAD^+^-dependent signaling pathway. However, whereas there is no doubt about the involvement of caspases in cell body destruction, their role in axonal and synaptic degeneration is still an open question.

We therefore decided to evaluate the synapto-protective potential of both NAD^+^ and z-VAD-fmk, a broad-spectrum caspase inhibitor. The cultures were pretreated for one hour with z-VAD-fmk (50 µM) (Fig. 4c,h) or for 24 hours with NAD^+^ (5 mM) (Fig. 4e,j). Cultures pretreated with NAD^+^ and subjected to axotomy retained 65% of the initial number of synapses whereas non-pretreated cultures retained 25% (Fig. 4k). In contrast, no significant synapto-protection was found in cultures pretreated with z-VAD-fmk (Fig. 4k). We also investigated the protective potential of resveratrol on synapses. As resveratrol, an activator of Sirtuins (a group of NAD^+^ dependent histone deacetylases) has been shown to have a similar protective effect on axons to NAD^+^ (Araki et al, 2004), we decided to evaluate its protective potential on synapses. However, in our experimental conditions, pretreatment with 20 µM resveratrol (1 h and 24 h) did not show any protective potential on synapses (Fig. 4b,g,k).

Finally, actin being a key component of pre and post-synapses which controls different aspects of synaptic physiology, we included a Rho Kinase inhibitor (Y27632) [24]. Interestingly, a strong synapto-protection was obtained with the Rho-kinase inhibitor (10 µM, 1 h pretreatment) with 75% of synapses preserved, indicating that ROCK may play an important role in presynaptic stability under stress. Our experimental setup thus successfully demonstrated that cortical axotomy induces synaptic disconnections preceding axonal degeneration and that NAD^+^ and Rho-kinase inhibitor have a specific protective effect against this cortico-striatal disconnection.

## Discussion

This tri-Compartiment (3C)-microfluidic system for neural network reconstruction and pathological process modeling is a good example of the interest of microfluidic for neurosciences. What neuroscientists need are sophisticated but easy to handle biological models to help solve complex neuro-scientific issues [20], [21]. Due to the inherent complexity of the organization of the brain, many questions regarding developmental issues, neuronal activities in complex networks and the mechanisms of neurodegeneration in either acute or neurodegenerative diseases remain unanswered. The lack of relevant but simplified models of neuronal networks has slowed down the development of efficient drug therapies which protect synapses and prevent network dysfunctions. Here we show that this new structured network integrating different neuron types that reproduce essential properties of neuronal communication is easy to handle and greatly facilitates the evaluation of synapto-protective drug potency.

Apoptosis is generally accepted to be a key event triggering neuron death in late-onset neurodegenerative diseases, yet their role in axonal and synaptic degeneration is unclear. Although caspases 3 and 9, which are key effectors of the apoptotic pathway, can be activated locally in synaptic terminals and neurites, resulting in local functional and morphological modifications [25], [26], they do not seem to play an active role in axotomy-induced axonal degeneration [27], [28]; see review [29], [30], [31]. Although it has been postulated that modest synaptic caspase activation might be involved in local non-apoptotic mechanisms such as long term depression [25], [26], our findings showing that caspase inhibitors failed to protect synapses after acute axonal injury further suggest that caspase activation is not a crucial mechanism in the synaptic degeneration process. Our findings are in line with the data showing that caspases are not involved in Wallerian degeneration processes, where it is rather NAD+ signaling which is involved. Indeed, the WldS gene was shown to confer a strong axo and synapto-protective effect at the neuromuscular junction through an age-dependent process. This gene mediates its effects partly through its nicotinamide monucleotide adenylTransferase-1 NMNAT1 moiety, an NAD+ producing enzyme [32]. Interestingly, in the CNS, the WldS gene delays nerve terminal degeneration in the striatum following ablation of the ipsilateral cerebral cortex [33]. Here we show that exogenous NAD+ strongly protects synapses after axonal trauma and confirm the importance of NAD+ signaling pathways in synaptic maintenance. However, both the targets of NAD+ and its site of action remain elusive. NAD+ is a molecule involved in many reactions and cellular functions including energetic metabolism, mitochondrial functions, and antioxidant defenses. It has long been postulated that NAD+ exerts axo-protective effects through nuclear signaling [34] but recent experiments have demonstrated that extra-nuclear NAD+ producing enzymes have the same effect [35]. Thus, although the mechanisms of NAD^+^ synapto-protective effects remain enigmatic, our data suggest that synaptic application of NAD+ may act locally to protect synapses after acute axonal injury. Finally, the results we obtained with caspase inhibitors and exogenous NAD+ are in line with data gathered from *in vivo* models, and therefore ensure the reliability of our evaluation assay. This encouraged us to assess the synapto-protective properties of two compounds targeting pathways potentially involved in synaptic collapse.

Among NAD+ potential targets, sirtuins represent attractive candidates. They are NAD+ dependent class III histone deacetylase involved in controlling longevity [36]. Sirtuin 1 (SIRT1) has previously been shown to mediate the axo-protective effect of WldS protein on axotomy [34]. Resveratrol that activates several sirtuins through indirect signaling pathways involving both AMPK activation and NAD+ production [37], [38], has been shown to have a similar axo-protective effect, once applied to entire neurons [34]. However, these results were not confirmed in another degenerative paradigm [39], and its effect on synapse is not known. It was therefore tempting to assess whether or not local (synaptic and axonal) application of resveratrol could delay synaptic destruction in our paradigm. The failure of synaptic application of resveratrol to directly protect synapses contrasts with the NAD+ protective effect. This may be due to the fact that resveratrol targets (either direct or indirect) are not expressed or enriched enough in presynaptic terminals to trigger a strong protective effect. This does not, however, rule out the possibility that resveratrol may exert a synapto-protective effect through long distance signaling such as nuclear SIRT1 activation, for example.

Finally, we wanted to see whether molecules which interfere with actin dynamic could slow down presynaptic collapse triggered by cortical fiber axotomy. In previous work we and others have shown that axotomy of cortical axons triggers a rapid collapse of actin filipodia in unconnected axons [16], [40], [41]. Pre and post synaptic elements are greatly enriched in actin networks and are virtually devoid of tubulin cytoskeleton. Actin dynamic controls most of the synaptic physiology. Among the effectors that control actin polymerization/depolymerization in neurons, Rho GTPase and Rho Kinase (ROCK), a group of serine/threonine kinases, are important players [42]. While the role of Rho GTPase in controlling dendritic and synaptic dynamic is becoming clearer, little is known about the role of Rho Kinase in synapses. ROCK signaling was recently involved in mediating growth cone collapse triggered by chemo-repulsive cues during brain development [43] and in controlling the readily releasable pool of synaptic vesicles [44]. Our results demonstrating that Y27632 is a potent inhibitor of presynaptic collapse show that ROCK are early effectors mediating destructive events involved in synaptic dismantlement. ROCK 2 was recently isolated in a proteomic and genomic screen aimed at isolating molecular effectors involved in synaptic degeneration [45]. It is thus tempting to propose that inhibitors of ROCK could represent interesting pharmacological compounds for further translational development.

### Conclusions

Microfluidic devices have facilitated investigation in biology in new ways, thanks to the structuring of the *in vitro* model, micro-environment control, and design flexibility. This work, which follows the trend of “organ on chip” research, shows that microfluidic platforms for nerve pathway reconstruction are useful tools for the study of both developmental and degenerative processes at several levels, ranging from sub-cellular compartments to integrated neuronal networks. Here we show that the use of this new sophisticated but still simple to handle *in vitro* reconstructed neuronal network not only allows basic biological questions to be addressed but also represents a valuable and relevant tool for fast screening of synapto-protective drugs. Using this strategy, it has been possible to reveal for the first time that Rho kinase may represent interesting targets to delay synaptic collapse after injury. If this could speed up and improve the reliability of the validation and characterization stages when developing therapeutic drugs, this would already be a great step forward.

## Supporting Information

Figure S1 **Automated quantification of synapses with SynD software.** Synaptic disconnection was assessed by counting v-GLUT1 clusters affixed to MAP-2 positive striatal dendrites. Images were all obtained with the same acquisition parameters and were similarly processed with ImageJ software before being used for quantification. (a) v-GLUT1/MAP-2 merge used for quantification using SynD software.(b) Automatic soma detected using thresholding followed by morphological opening and closing. (c) Dendrites detected using steerable filters. A cost function evaluates each pixel around the current neurite mask, and those with a cost lower than a specified threshold are added to the mask. The cost function takes into account how neurite-like the neighborhood around the candidate pixel is (i.e. ideally a bright line segment surrounded by dark regions on either side), and if the candidate pixel is in the direction of the already detected parts of the neurite. (d) The detected neurite mask overlaid on the v-GLUT1 channel, only the parts located either within the neurite mask or a small neighborhood around it are used for synapse detection. (e) Synapse regions are detected by thresholding the v-GLUT1 channel; putative centers are located by deconvoluting the image.
(EPS)
